# Loss of Fine Motor Dexterity and Reduced Oral Hygiene 15 Years After Diagnosis of Multifocal Motor Neuropathy with Screw-Retained Implant-Supported Rehabilitation: Literature Review and Case Report

**DOI:** 10.3290/j.ohpd.b5828022

**Published:** 2024-11-18

**Authors:** Pascal Grün, Florian Pfaffeneder-Mantai, Justin Graf, Werner Lill, Dritan Turhani

**Affiliations:** a Pascal Grün Doctor of Medicine in Dentistry, Senior Doctor, Center for Oral and Maxillofacial Surgery, Department of Dentistry, Faculty of Medicine and Dentistry, Danube Private University, Austria. Study conception and design; data collection, analysis and discussion, draft, discussion and finalisation of the manuscript.; b Florian Pfaffeneder-Mantai Doctor of Medicine in Dentistry, Senior Doctor and Deputy Medical Director, Center for Oral and Maxillofacial Surgery, Department of Dentistry, Faculty of Medicine and Dentistry, Danube Private University, Austria; Division for Chemistry and Physics of Materials, Department of Medicine, Faculty of Medicine and Dentistry, Danube Private University, Austria. Study conception and design; prevention and care for dental treatment.; c Justin Graf Student of Medicine in Dentistry, Center for Oral and Maxillofacial Surgery, Department of Dentistry, Faculty of Medicine and Dentistry, Danube Private University, Austria. Study conception and design; data collection; graphical representation.; d Werner Lill Doctor of Medicine, Private Practice for Periodontology and Implantology, Vienna, Austria. Study conception and design, analysis and discussion of data.; e Dritan Turhani Doctor of Medicine, Professor and Head, Centre for Oral and Maxillofacial Surgery, Center for Oral and Maxillofacial Surgery, Department of Dentistry, Faculty of Medicine and Dentistry, Danube Private University, Austria. Study conception and design, data collection, medical examination and treatment of the patient, draft, discussion and finalisation of the manuscript.

**Keywords:** case report, mouth rehabilitation, multifocal motor neuropathy, oral health, peri-implantitis, review

## Abstract

**Purpose:**

Multifocal motor neuropathy (MMN) is a rare immune-mediated neuropathy causing progressive, asymmetric weakness without sensory loss. Long-term administration of intravenous (IVIg) or subcutaneous (SCIg) immunoglobulins is the primary therapeutic approach. Despite this, therapy failure can result in a decline in strength, particularly in the hands, impacting daily activities. This review and case report presents the current literature on this complex dental and medical topic and explores the novel use of dental implants for full-mouth rehabilitation in MMN patients undergoing long-term IVIg therapy.

**Materials and Methods:**

A patient with MMN underwent 15 years of long-term treatment with intravenous immunoglobulin (IVIg), starting with an initial dose of 0.4 g/kg for 5 days every 4 months in 2008. The maintenance dosage of 0.2 g/kg as a single dose every 3 months was established as a long-term therapy. In 2017, the patient received a maxillary and mandibular complete-arch implant-supported prosthesis.

**Results:**

MMN showed no progression until the IVIg interval was extended to every 4 months in 2022. Significant deterioration in dental health resulted from a loss of right-hand dexterity, which affected toothbrush use and interproximal brushing, resulting in poor oral hygiene. Dental hygiene and dental health, which were not optimal anyway, were considerably worsened by the loss of dexterity in the right hand, which impaired the use of the toothbrush and the cleaning of the interdental spaces.

**Conclusion:**

Dental implants are a well-established treatment for edentulous patients, but their success in those with MMN requires careful consideration of oral health practices. Effective maintenance protocols and optimised prosthetic designs are crucial for long-term implant therapy success in MMN patients. Peri-implant diseases pose risks influenced by various factors. In the case of MMN and its neurological considerations, implications for dental implant provision warrant further exploration, considering clinical symptoms, therapy, and potential progression.

Multifocal motor neuropathy (MMN) is an acquired immune-mediated neuropathy with distinct clinical and electrophysiological manifestations.^13,20^ It was first described in 1986 in a patient with chronic, asymmetrical, distal motor neuropathy without sensory loss, who had initially myokymia and fasciculations without atrophy.^40,82^ MMN is a rare disorder with a prevalence ranging from 0.29 to 0.70 per 100,00013,40 and a male-to-female ratio of 2:13 or higher.^40^ The age of onset ranges from 20 to 70 years, and most patients develop the disorder between 25 and 50 years of age.^3,94^

In general, MMN is characterised by slowly progressive, asymmetric, predominantly distal weakness of one or more limbs with no objective loss of sensation.^18,57,70,71^ Important differential diagnoses are amyotrophic lateral sclerosis (ALS), chronic inflammatory demyelinating polyneuropathy (CIDP), and hereditary neuropathy with liability to pressure palsies (HNPP).^4,16^ The diagnosis of MMN requires slowly progressing, focal, asymmetric limb weakness with the involvement of at least two motor nerves without sensory impairments except for minor vibration sense abnormalities in the lower limbs.^21,40^ Most patients develop a progressive decline in strength, especially in the hands and arms, which can lead to difficulties in performing even simple daily tasks such as writing, washing, or dressing.^49^ Two main lines of evidence suggest that MMN is an immune-mediated disorder. First serum immunoglobulin M (IgM) ganglioside-monosialic acid (GM1) antibodies are found in 30–80% of people with MMN.^102,105^ Second, various immunomodulatory and immunosuppressive treatments have been used, with the most promising therapeutic strategy being based on the long-term use of intravenous immunoglobulins (IVIg).^4,50^ IVIg is a preparation of highly purified immunoglobulins, predominantly comprising IgG subclasses.^100^ Newest reports have shown that long-term treatment (up to 96 months) with subcutaneous immunoglobulins (SCIg), previously stabilised with IVIg therapy, has been as effective as IVIg in patients with MMN.^38^

In some patients with MMN, long-term IVIg treatment causes generalised periodontal destruction,^22^ oral lichen planus,^37^ or necrotising ulcerative periodontitis.^11^ At present, literature evaluating the use of dental implants in patients with MMN is lacking. This gap underscores the importance of investigating potential reasons for the occurrence of peri-implantitis in this patient population.^15^

Dental implants represent an established and widely recognised treatment modality for replacing missing teeth in edentulous patients.

Another important aspect of implant dentistry is its impact on patients’ quality of life. Duong et al investigate oral health-related quality of life in patients rehabilitated with fixed and removable implant-supported prostheses. Their research shows that while both types of prostheses improve patients’ quality of life, fixed prostheses are generally associated with higher patient satisfaction and fewer functional limitations. These findings are crucial for patient counselling and treatment planning.^30^

Strong evidence suggests an increased risk of peri-implantitis among individuals with poor personal and professionally administered oral hygiene practices, as well as those with a history of periodontitis.^67,68,84^ The aetiology and pathophysiology of peri-implant diseases (PIDs) remain under investigation, with several risk factors/indicators advocated as potential contributors to peri-implant tissue breakdown.^34,87^ These include smoking,^24,80^ diabetes mellitus,^48^ periodontitis,^90^ limited/lack of supportive peri-implant care,^47^ inadequate personal biofilm control,^77^ reduced peri-implant keratinised mucosa (PIKM),^97^ and some mechanic^78^ characteristics of the implant-supported restoration design.^84,89^ Furthermore, genetics, stress, diet, and other lifestyle habits may be considered potential risk factors for PID.^9,60^

Ramseier reviews the diagnostic tools currently used in periodontics and implantology, emphasising the importance of early detection and regular follow-up to maintain periodontal health and implant longevity. Ramseier describes specific diagnostic measures such as radiographic monitoring, probing depth measurements, and biomarkers that help clinicians assess the condition of peri-implant tissues and detect early signs of disease progression.^76^

Finally, implant loss is a major problem in implantology, with Tomasi and Derks examining the aetiology, occurrence, and consequences of implant failure.^98^ Their review highlights factors such as biomechanical overload, infections, and systemic health conditions that may contribute to implant loss. The authors also discuss the long-term consequences of implant failure for patients, including limited function, aesthetic challenges, and emotional distress.

A new trend in the assessment of periodontitis and peri-implantitis patients is the inclusion of patient-related outcomes and patient-reported experiences, as reviewed by Arunyanak et al. Their study highlights the growing importance of the patient perspective in clinical decisions and suggests that PROs and PREs provide valuable insights into patient satisfaction, comfort and overall quality of life. These measures allow clinicians to evaluate treatment success not only from a clinical perspective but also from the subjective experience of patients.^6^

In the context of neurological disorders, such as MMN, the impact of oral health (OH) behaviour on peri-implant health and disease also plays a crucial role. OH is the process of cleaning the hard and soft tissues of the oral cavity (teeth, gums, and tongue), dental implant-supported prostheses, oral appliances, and dentures.^103^ Consistent professional maintenance and the standard of the patients’ home care remain key factors for avoiding plaque accumulation and peri-implant tissue inflammation and preventing biological complications.^12,91,93^ The risk of diseases and disabilities that can affect OH-related performance increases with age.^65^

The effectiveness of manual toothbrushing generally depends on several factors, including brushing movements and hand-motor function.^7,29,31^ In particular, impaired finger or hand joint function can influence the extent of dental biofilm formation.^32,73,75^ A decrease in hand grip strength or manual dexterity favours the accumulation of pathogenic dental biofilm in independent older adults.^2^ In this patient population, implant-supported full-mouth restorations are particularly prevalent for comprehensive oral rehabilitation, especially in completely edentulous individuals.^78^

Challenges in maintaining optimal OH are frequently observed in patients with neurological diseases^64^ such as ALS,^63^ Alzheimer’s disease (AD),^36^ Down syndrome (DS),^58^ and post-stroke conditions.^41^ So far, no such experiences have been described in patients with MMN. This case report presents the first description of a patient with MMN undergoing prolonged IVIg treatment who experienced a decline in fine motor dexterity following therapy reduction. This initiated the manifestation of clinical and radiological peri-mucositis signs 6 years after the placement of a full-mouth screw-retained implant-supported metal-resin prosthesis.

## MATERIALS AND METHODS

**Fig 1:**
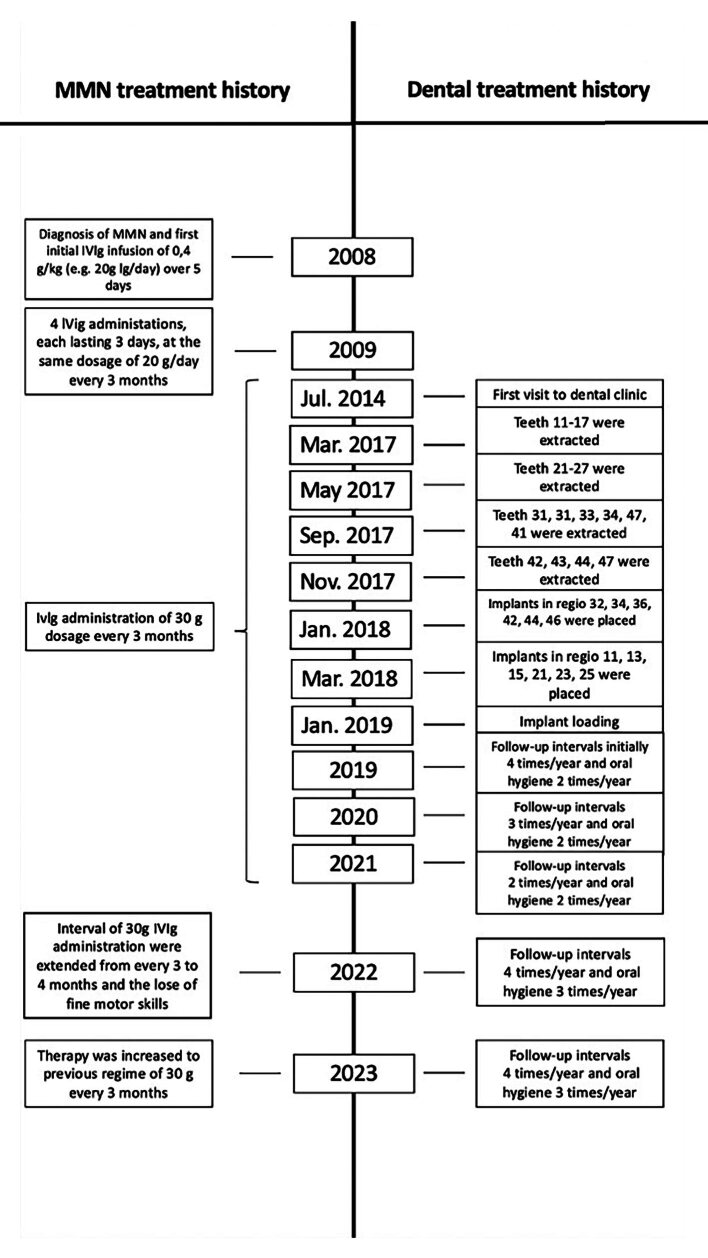
Timeline of MMN and dental treatment history over 15 years. IVIg, intravenous immunoglobulins; MMN, multifocal motor neuropathy.

We present the case of a 75-year-old Caucasian male patient diagnosed with MMN receiving long-term IVIg treatment over a period of 15 years. In January 2008, at the age of 59, the patient first noticed weakness in the left lower extremity. His genetic profile, as well as his psychological, family, and drug history, were unremarkable, and he was a nonsmoker. By April 2008, weakness was also noticed in the left middle finger and a general lack of strength in the right hand, with occasional cramps. No sensory disturbances were observed. The patient’s neurological status was unremarkable, except for a slightly stronger tremor in the right hand compared to the left. The main notable findings were conduction blocks in the forearm region of the left ulnar nerve and around the right radial nerve at the elbow. Considering the demonstrated multifocal conduction blocks outside anatomical entrapment sites and elevated anti-GM1 antibody levels, the primary assumption was MMN. Abdominal sonography and the assessment of beta-2 microglobulin levels were also planned, but the patient decided against it. In December 2008, the patient received the first planned IVIg administration. Symptoms remained unchanged, with occasional stumbling due to the left leg and infrequent cramps in the right hand after exertion. The initial IVIg infusion of 0.4 g/kg, eq. 20 g Ig/day, was administered over 5 days without any adverse effects. The patient mentioned receiving regular internal medical care for IgM paraproteinemia. The patient was discharged in good general condition and received between April 2009 and April 2010 four additional IVIg administrations, each lasting 3 days at the same dosage of 20 g Ig/day, all of which were well-tolerated. He reported symptom reduction at each appointment, with fine motor skills in the right hand becoming comparable to those in the left hand. Given the subjective improvement, electrophysiological progress, and excellent infusion tolerance, the intervals were extended from approximately every 4 to every 6 months. In May 2011, during the patient’s last inpatient visit, weakness was mainly reported during intense physical exertion. The neurological assessment at admission revealed pronation and slight lowering of the right upper extremity, right-sided bradydiadochokinesia, and the previously identified drop of the right forefoot. The patient showed good IVIg tolerance, received a 30 g dosage on the first day, and was discharged the next day. Subsequently, 30 g IVIg were administered as outpatient infusions every 3 months, eliminating the need for hospitalisation (Fig 1).

**Fig 2:**
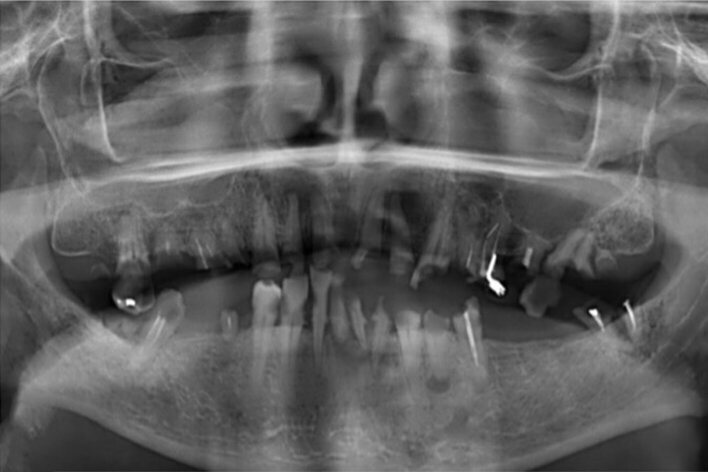

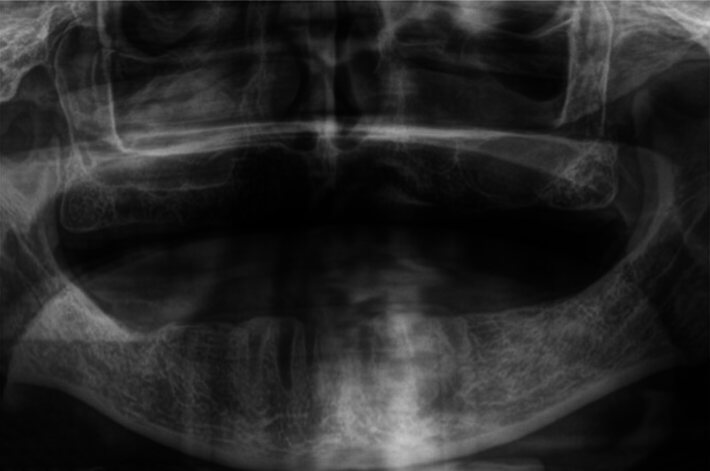

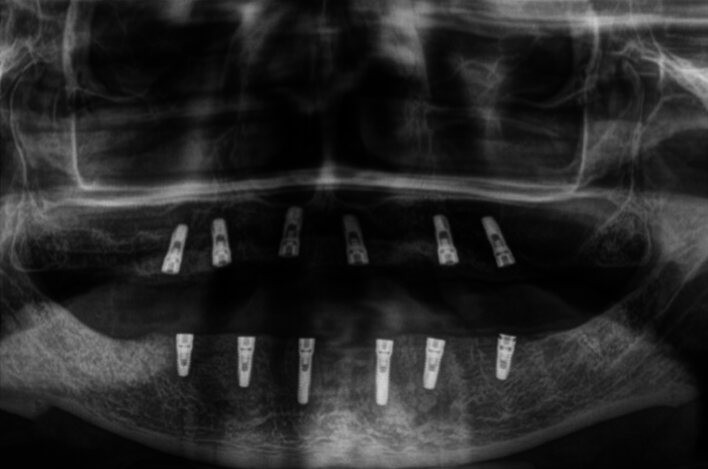

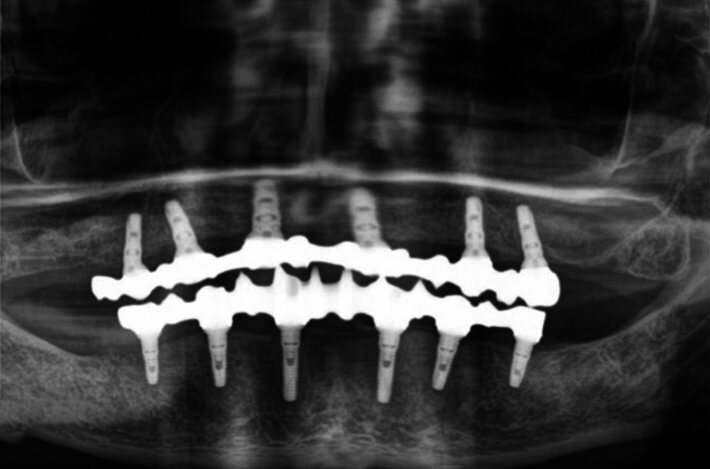
(a) OPT at the initial appointment at the dental clinic. (b) OPT after serial extractions in the mandibular and maxillary jaws. (c) OPT after insertion of six dental implants in the mandibular and maxillary jaws. (d) OPT 3 years after prosthetic rehabilitation.

In July 2014, the patient was referred to our dental outpatient clinic because of pronounced tooth pain and increased tooth mobility that occurred after the start of the immunoglobulin therapy. Upon clinical examination, teeth 17, 13, 12, 11, 21, 23, 25, 27, 35, 33, 32, 31, 41, 42, 43, and 47 and root residues 16, 15, 14, 22, 24, 26, 38, 34, and 44 were found to be severely carious. In addition, the patient showed dental mobility in several elements along with a considerable accumulation of gross calculus. The radiographic examination (orthopantomogram (OPT)) of the present teeth showed generalised and pronounced bone loss (Fig 2a).

Due to personal reasons, the patient adopted a wait-and-see approach. In December 2016, two alternative therapy plans were discussed: a prosthetic restoration with total dentures or an implant-supported full-mouth restoration. The patient ultimately chose the implant-supported full-mouth restoration. In four appointments from March to September 2017, all teeth and root fragments were successfully extracted under local anaesthesia.

All extraction sockets were thoroughly treated with aggressive curettage, preserved with allogeneic bone substitute material (Puros, ZimVie, Colorado, United States), and covered with a pericardium membrane (CopiOs, ZimVie). The intra- and postoperative course was uncomplicated (Fig 2b). In two separate appointments in January and March 2018, a total of 12 BEGO S-Line (BEGO Implants, Bremen, Germany) implants, each with a diameter of 3.25 mm, were successfully placed. After local anaesthesia using articaine 4% with 1:200,000 adrenaline (3M ESPE, St. Paul, Minnesota, USA), the placement of 12 dental implants was planned under standard protocol. All implants were placed with minimal torque above 30 Ncm by forming mucoperiosteal flaps and adhering to standard drilling protocols. Implants with 10 mm length were placed in regions 11, 13, 15, 21, 23, and 25; implants with a length of 13 mm in regions 32 and 42; implants with a length of 10 mm in regions 34 and 44; and implants with a length of 8.5 mm in regions 36 and 46. In region 42, an augmentation procedure was performed with a simultaneous autologous bone graft covered with a pericardium membrane (CopiOs). Additionally, a bone-splitting technique was employed in regions 21–25 prior to successful implant placement. To enhance the stability of the buccal bone structure, bone graft material (BEGO Oss), covered with a pericardium membrane (BEGO collagen membrane, both BEGO Implants) was applied (Fig 2c). To close the flaps, 6-0 Prolene (Johnson & Johnson, New Brunswick, NJ, United States) monofilament interrupted sutures were used. The patient was provided with postoperative oral care instructions, which included rinsing the oral cavity with a 0.2% chlorhexidine gluconate mouthwash solution (GlaxoSmithKline, Baar, Switzerland) twice daily for one week and using an extra-fine toothbrush to clean the temporary restorations. The patient received an antibiotic (1 g amoxicillin/clavulanate; GlaxoSmithKline) twice a day for 5 days. For pain control, the patient was prescribed 100 mg of mefenamic acid (Pfizer, Vienna, Austria). The patient was advised to minimise trauma at the surgery site; a specific diet was not recommended. The sutures were removed 10 days after surgery. Subsequent postoperative follow-up visits revealed no complications. The patient was provided with a transitional prosthesis for 13 months until the implants could be loaded.

Due to the high primary stability, the implants could have been loaded earlier, but the patient could not coordinate the appointments for the prosthetic restoration due to medical hospitalisation.

Before fabricating the definitive restorations, prototypes were made during the design and testing phase, which was used to evaluate aesthetics, phonetics, function, and hygiene. The prototypes (screw-retained resin) were worn by the patient for 12 weeks to assess, among other things, the patient’s ability to practice proper hygiene. As no difficulties were encountered, the design was transferred unchanged to the final implant-screw-retained titanium-base resin-veneered prosthesis in January 2019. To enable careful monitoring of patient compliance and considering that most of the bone remodelling occurs within 12 to 16 months after prosthetic rehabilitation, follow-up appointments were performed four times in the first year (2019), three times in the second year, and twice in the third and all subsequent years. Regular professional cleanings and careful oral hygiene instructions were carried out in constant cooperation between a certified prophylaxis assistant and an experienced dentist. The patient was always instructed to use interdental brushes and dental floss during daily oral hygiene to ensure optimal plaque control around the implant sites. Probing depths and probing bleeding were measured at each session.

In each visit, the patient’s ability to maintain proper oral hygiene was demonstrated, and abnormalities were not detected (Fig 2d). The patient’s adherence to the recommended treatment and oral hygiene protocols was satisfactory. The clinical parameters for implant monitoring were thoroughly checked at each follow-up examination. The modified bleeding on probing remained consistently below 10% and the plaque index was within an acceptable range at 15%. The probing pocket depths around the implants were stable at 3 mm, indicating healthy peri-implant tissue.

## RESULTS

**Fig 3:**
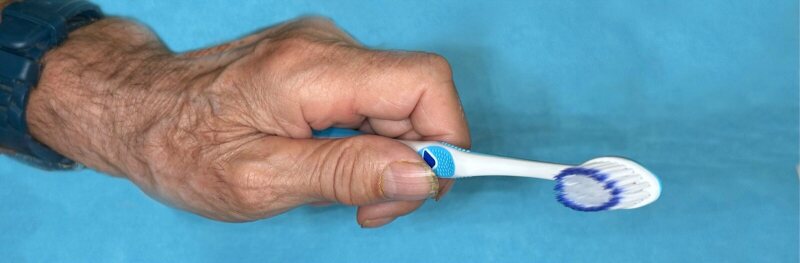

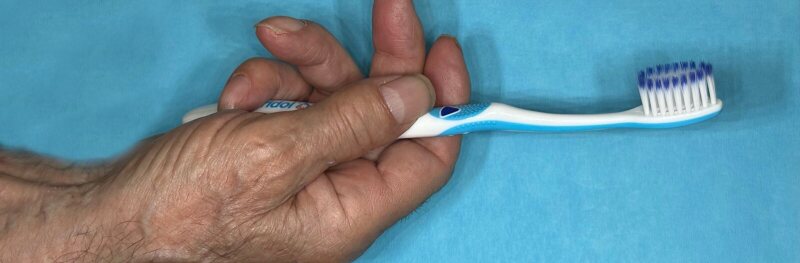

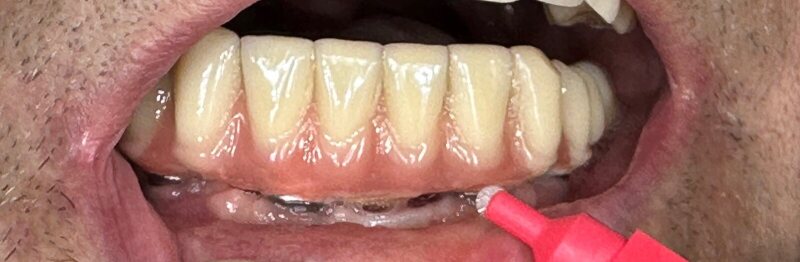
(a) Oral hygiene had to be carried out with the left hand due to the loss of fine motor skills in the right hand. (b) The patient was unable to use a toothbrush correctly with the right hand. (c) Clinical situation after reduction of denture bases to simplify oral hygiene.

In January 2022, the health insurer extended for unclear, presumably economic and medically unjustifiable reasons, the interval of the 30 g IVIg administrations from every 3 to every 4 months. After this frequency reduction, the patient encountered a loss of fine motor skills and hand grip strength in the right hand. From a dental point of view, this rendered him unable to hold the toothbrush or interproximal brushes, let alone exert pressure, leading to a serious setback in maintaining proper oral hygiene. Due to this deterioration, the IVIg administration frequency was reverted in January 2023 to 30 g every 3 months. However, the neurological symptoms in the fingers did not resolve, and the reduced oral hygiene could not be improved. The right-handed patient was unable to hold a toothbrush, dental floss, or interdental brush with his right hand, and the weaker left hand could only use a toothbrush, which did not allow thorough plaque removal (Figs 3a and 3b).

The interval of dental follow-ups was promptly adjusted from two to four times per year to assess and guide his ability to maintain proper oral hygiene. Well-established maintenance protocols were required to minimise the risk of severe biological and mechanical complications of his full-mouth screw-retained implant rehabilitation. Although many protocols over the past decades have suggested not to remove implant-supported prostheses for maintenance and cleaning purposes, we removed the prostheses at follow-up visits for thorough inspection and implant cleaning once a year. To simplify oral hygiene, the denture bases have been reduced in size so that they have no contact with the mucosa and the hygiene space between the denture base and the mucosa is at least 1–2 mm (Fig 3c).

**Fig 4:**
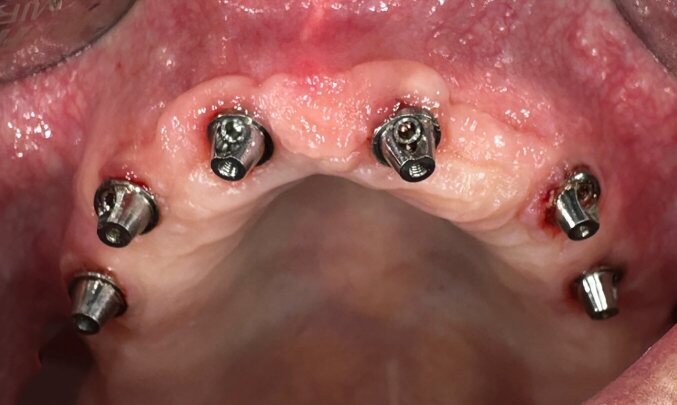

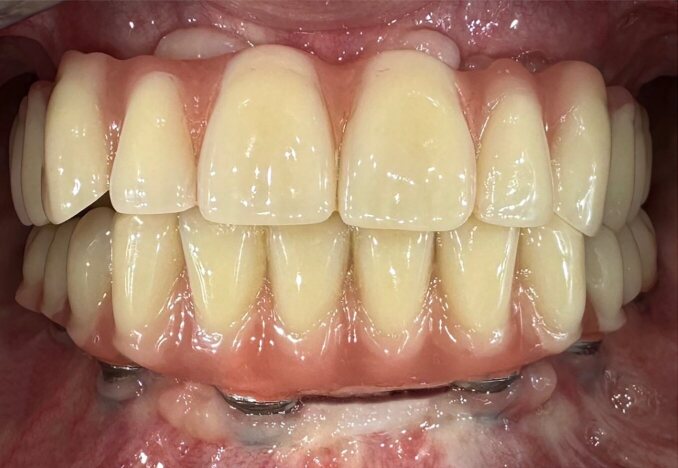

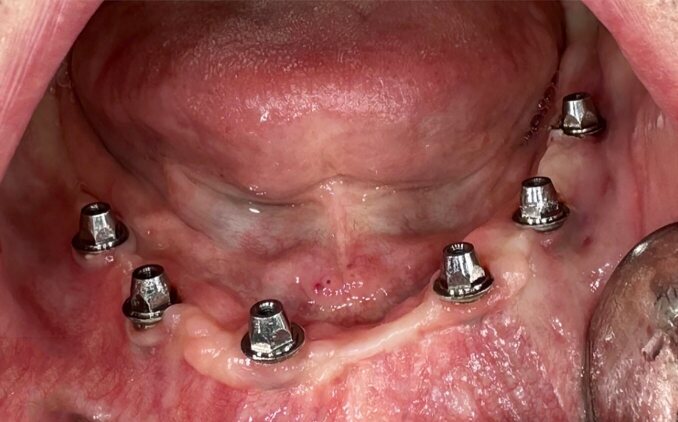
Increases in the frequency of check-ups improved oral hygiene, and no inflammatory processes near the implants were detected during check-ups.

After increasing the check-up frequency and supporting oral hygiene, inflammatory processes around the implants were not detected during check-ups in May and September 2023 after a thorough clinical and radiological examination (Fig 4).

## DISCUSSION

Dental implants are a common solution for prosthetic restoration in patients with partially or fully edentulous arches.^17^ A prerequisite for the long-term success and survival of dental implants is the sufficient amount and quality of bone surrounding the implant. Peri-implantitis is a key factor that negatively influences the bone surrounding the implant causing bone resorption and ultimately leading to decreased attachment between the bone and the implant.^90^ PIDs, pathological inflammatory disorders induced by biofilm accumulation, start in the soft tissue compartment and progress in an accelerated nonlinear pattern to the underlying peri-implant bone.^35,46,90^

In the 2017 World Workshop on the Classification of Periodontal and Peri-Implant Diseases and Conditions,^14^ PIDs were classified into peri-implant mucositis, peri-implantitis, and soft- and hard-tissue deficiencies around dental implants.^96^

Biofilm is a sticky microbial community with more than 700 different species of bacteria attached to salivary glycoproteins.^28,31,66,107^ The easiest and most effective method to remove dental biofilm is toothbrushing.^25,31,66,107^ However, the effectiveness of manual toothbrushing generally depends on several factors, including brushing movements and hand-motor function.^7,29,31^ In particular, impaired function of the fingers or joints of the hand affects the extent of dental biofilm formation.^32,73,75^

Factors of medical history influence the PID risk in addition to the eight parameters proposed in the Implant Disease Risk Assessment (IDRA) by Heitz-Mayfield in 2020^45^: (1) history of periodontitis; (2) percentage of sites with bleeding on probing; (3) prevalence of probing depths greater than 5 mm; (4) periodontal bone loss in relation to the patient’s age; (5) periodontitis susceptibility; (6) supportive periodontal therapy; (7) proximity of the restoration margin to the bone crest; and (8) implant prosthesis-related factors are factors of medical history as well.^42,90^ Patients with a higher risk should be followed up more frequently, and patient education is important to improve patient awareness for disease prevention.^45^

Emerging risk factors/indicators for peri-implantitis, which were not included in the IDRA tool, are for example smoking,^79^ glycaemic index, genetic and epigenetic susceptibility, implant position,^42^ peri-implant soft tissue,^27^ type of prosthetic connection, and occlusal strength.^42^ Additional factors may affect crestal bone stability, such as implant surface characteristics and implant neck/platform designs, or disorders associated with systemic chronic inflammation, such as diabetes,^48^ cardiovascular disease, and rheumatoid arthritis.^45^ To our knowledge, the literature has neither described MMN as a risk factor for implants nor the oral consequences of disease progression.

Individuals affected by MMN present clinically with subacute to chronic progressive asymmetric muscle weakness. Initial symptoms may include unilateral wrist drop, finger weakness, or foot drop. Additionally, sensory symptoms like pain and tingling are not uncommon. Physical examination findings reveal weakness in distal muscles corresponding to the distribution of the affected motor nerves. For example, with radial nerve involvement, the wrist and finger extensors are more affected than the triceps. Muscles of the same myotome may be spared if innervated by a different nerve. Muscle atrophy may become apparent in later disease stages, yet is often disproportionately mild compared to the weakness observed.^4,106^

Various immunomodulatory and immunosuppressive treatments have been suggested, but the treatment of choice is the long-term administration of IVIg infusions (evidence level I).^5^ IVIg is a preparation of highly purified immunoglobulins from a large pool of plasma from healthy human donors. Human IVIg contains biologically active IgG and trace amounts of IgA, IgM, CD4, CD8, and human leukocyte antigen molecules.^95^ Since IVIg preparations are heterogeneous, it is difficult to determine their exact mechanism of action. It is widely postulated that the efficacy of IVIg therapy is linked to its ability to block Fc receptors, eliminate autoantibodies, modulate cytokine synthesis, inhibit complement, and mediate Fas–Fas ligand interactions.^33^ The European Federation of Neurological Societies (EFNS) guidelines recommend 2 g/kg of IVIg as the first-line treatment given in several doses over 2–5 days.^8^

In most patients with MMN, IVIg effects last only a few weeks, and maintenance treatment with periodic IVIg infusions for extended periods of time is often indicated.^56,101^ However, the effectiveness of the treatment often diminishes over prolonged use, requiring an increase in dosage, a higher frequency of administration, or both.^43,50,59,74,83^ Optimising therapy through dose and interval adjustments to avoid end-of-dose worsening may promote disease stabilisation and long-term recovery.^38,54,104^ IVIg therapy may also improve muscle strength. Since weakness primarily contributes to disability in individuals with MMN, an increase in muscle strength, specifically grip strength, can serve as a valuable metric for evaluating an individual’s response to IVIg. Furthermore, disability improvement is the primary goal of treatment and the most important parameter in deciding whether to continue treatment. If the disease conditions do not interfere with activities of daily living, people are often only closely monitored without treatment due to minimal expected treatment benefits, high burden of regular IV fluid therapy, and high treatment costs. In most but not all individuals with MMN, discontinuation of IVIg therapy leads to a deterioration in muscle strength and disability. Only very limited evidence supports the use of SCIg as an alternative to IVIg maintenance therapy, and the evidence on adverse effects of IVIg compared to placebo or SCIg is very limited.^50^

In general, adverse reactions to IVIg therapy are usually minor and occur in no more than 10% of patients.^23^ The most common unwanted effect of IVIg use is acute hypersensitivity, but adverse effects can include headache, flushing, malaise, chest tightness, fever, chills, myalgia, fatigue, dyspnoea, back pain, nausea, vomiting, diarrhoea, blood pressure changes, and tachycardia.^23,72^ The aggravation of periodontitis is considered a new adverse effect of IVIg therapy, and it has been reported that oral conditions may deteriorate after starting IVIg therapy.^22^ Another mechanism related to adverse reactions in IVIg therapy is the formation of oligo- or polymeric IgG complexes that interact with Fc receptors and trigger the release of inflammatory mediators. The direct ligation of IgG to Fc receptors on immature osteoclasts can result in enhanced osteoclast generation and, ultimately, bone destruction.^23^

In our present case, the interval of IVIg therapy was increased from 3 to 4 months. This change caused the serious and irreversible loss of fine motor skills, leaving the patient unable to effectively hold or use a toothbrush or an interdental brush. Consequently, the patient was unable to maintain the required oral hygiene necessary for full-mouth rehabilitation. Without appropriate oral hygiene, the occurrence of peri-implant events cannot be averted.

The literature describes that the proportion of individuals with reduced grip strength is greater in individuals with periodontitis than in those without. In addition, their study confirms that the higher the hand grip strength, the lower the incidence of periodontitis. Thus, decreased hand grip strength may be a predictor of periodontitis in ageing adults. Hand function, especially manipulation skills, begins to decline in middle-aged individuals.^26^ Several previous studies, mostly involving older participants, highlighted the importance of hand function in the quality of oral care.^32^ One study reported that older adults with reduced manual dexterity or hand grip strength had a higher accumulation of mature dental biofilm, a causative factor in oral disease.^92^ Hashimoto et al describe that older adults aged 80 and over who had high hand grip strength had more teeth than those with low hand grip strength.^44^ Poor hand grip strength can lead to discomfort and premature hand fatigue when handling a toothbrush grip. Fatigue, in turn, reduces brushing time, brushing power, and brushing movements, ultimately leading to less efficient biofilm removal. Tailored oral hygiene regimens should also be provided based on individual hand grip strength.^53,55,86,99^ The adjunctive use of chemical antiplaque agents, probiotics,^51,69^ and anti-inflammatory agents^61^ offers clear benefits in reducing gingival indices in humans with gingivitis.^19^ Therefore, dental hygienists must continuously motivate and support adults with low hand strength to ensure optimal oral hygiene.^2^ For clinicians, it is important to keep in mind that the key to a successful outcome is ensuring pre-implant preparation and assessment, facilitating patient education, controlling periodontal disease, and establishing an ideal surgical and prosthetic treatment plan.^96^ It is also important that the prosthetic design of an implant restoration is closely related to the future peri-implant health. The concepts and restorative outcomes must be considered when planning implant treatment. The planning process should include considerations of the restorative contour and the cleanability of the prosthesis.^42^ Fixed implant rehabilitations should use tall abutments and prostheses completely free of tissue contact with 1–2 mm of hygiene space underneath.^1,88^ The design of embrasure spaces in implant restorations should allow for the effective insertion of interproximal brushes without causing mechanical trauma to the mucosal tissues.^39,62,85^ Without additional help and participation in dental monitoring and care during the recall procedure for this patient group, gradual deterioration of oral hygiene may inevitably occur, resulting in inflammation.^10^

**Fig 5:**
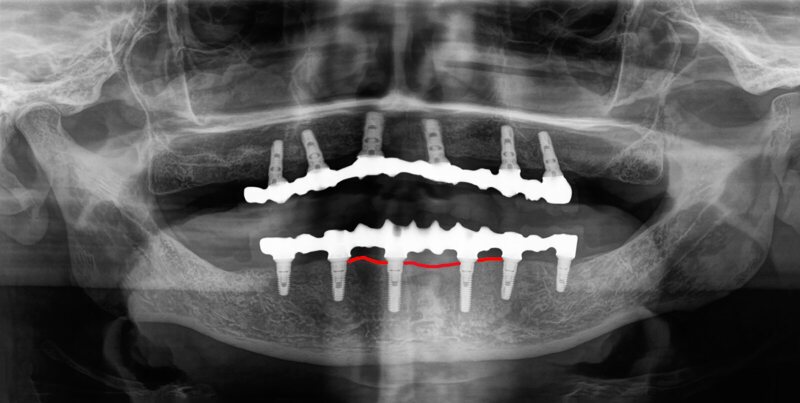

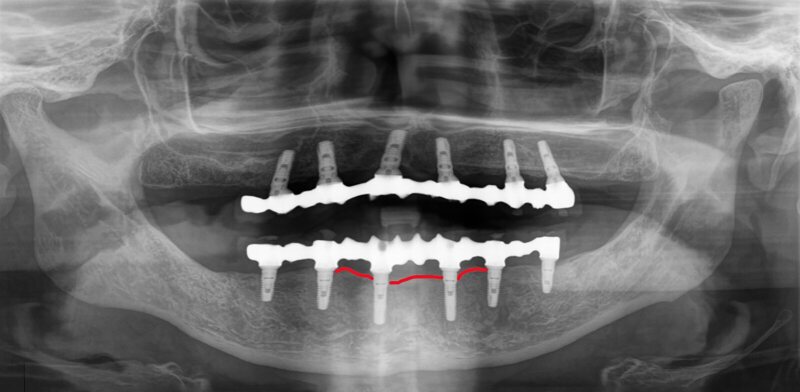
Radiographic follow-up with marked bone height from 01/2019 (a) and 09/2022 (b). The bone recession is clearly visible here.

**Fig 6:**
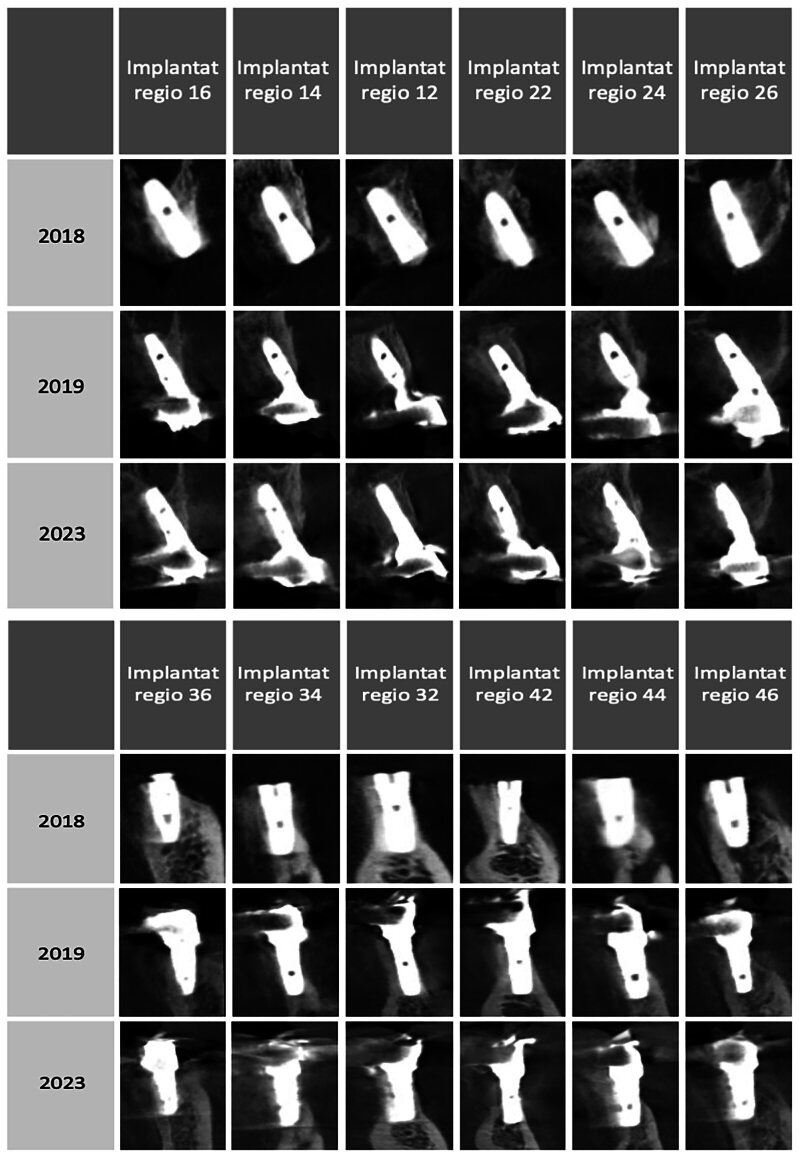
CBCT follow-up of individual implants.

Regrettably, we were not informed about our patient’s loss of fine motor skills and were therefore only able to diagnose the bone loss and deterioration of oral hygiene during radiographic check-ups (digital volume tomography [DVT]) and professional oral hygiene sessions (Figs 5 and 6). Therefore, we were unable to facilitate the early implementation of additional oral hygiene measures and dental examinations for the patient.

Considering that the implants have not exhibited any radiological bone loss over the last 5 years and that this coincided with the onset of fine motor skill deterioration within a year, our opinion is that oral hygiene may not have been adequately maintained.

Having natural teeth or fixed reconstructions in old age implies improved health and increased quality of life, but it also entails an increased risk of oral diseases, thus the need for high-quality oral care increases. This includes a high level of daily oral hygiene, regardless of whether this is undertaken by the older person independently or with assistance.

Considering that the primary aetiology of PIDs is bacterial plaque and that disease progression is a consequence of the host response to the biofilm formation, the therapy for peri-implant mucositis and nonsurgical therapy for peri-implantitis usually involves mechanical or chemical debridement of the dental implant surfaces. This treatment may or may not involve the adjunctive use of local antibiotics or antiseptics to decrease the bacterial burden.^52,81,96^

In summary, recent literature emphasises a multifaceted approach to periodontal and peri-implant health that includes advanced diagnostic measures, consideration of patient-reported outcomes, and a comprehensive understanding of risk factors and treatment strategies for peri-implant disease. These findings highlight the need for personalised, patient-centred care that addresses both clinical and psychosocial aspects of treatment outcomes.

This case report describes a patient with MMN who was deprived of the optimal IVIg dose for economic reasons. This reduction in medication dose resulted in a deterioration of the patient’s condition, leading to the irreversible loss of fine motor dexterity and grip strength. As a result, the patient was no longer able to maintain oral hygiene independently and adequately after a full-mouth reconstruction. Immediately after reducing IVIg therapy and the consequent loss of fine motor skills, the impact on dental health was considerable.

In the preoperative planning for patients with MMN, an interdisciplinary approach is therefore urgently indicated. After the insertion of dental implants, optimal MMN therapy must be guaranteed. Furthermore, drug-related adverse effects and worsening of typical MMN symptoms must be considered in the preoperative phase. For this reason, prosthetic planning should take into consideration that the prosthesis can be cleaned with reduced fine motor skills.

We thank the patient for permission to use his clinical data and Wiley Editing Services for editing. We thank Ditjon Bytyqi for the graphic design.

### Statements funding

This research received no specific grant from any funding agency in the public, commercial, or not-for-profit sectors.

### Conflict of interest disclosure

The authors have no conflicts of interest to declare.

### Patient consent statement

General informed consent was provided for the patient. Written informed consent was obtained from patient for publication of the details of his medical case and any accompanying images.

### Data availability statement

The data that support the findings of this study are available on request from the corresponding author. The data are not publicly available due to privacy or ethical restrictions.

### Statement of ethics

This study was approved by the Committee for Integrity and Ethics in Research of Danube Private University with approval number (DPU-EK/041).

## REFERENCES
